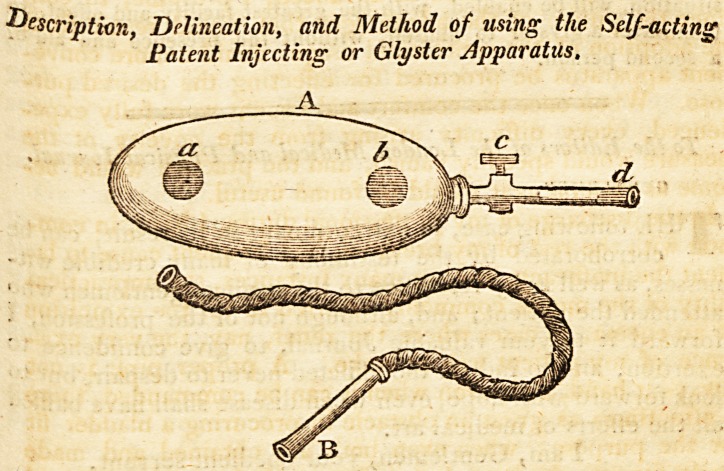# Practical Remarks on the Utility of Enemas, and on an Improved Method of Administering Them

**Published:** 1818-09

**Authors:** T. Machell

**Affiliations:** Member of the Royal College of Surgeons, and late Surgeon to the Truss Society for the gratuitous Cure of Ruptures, under the Patronage of his Royal Highness the Duke of Sussex.


### *For the London Medical and Physical Journal.*

*Practical Remarks on the Utility of Enemas, and on an improved Method of administering them.* By T. MACHELL, Member of the Royal College of Surgeons, and late Surgeon to the Truss Society for the gratuitous Cure of Ruptures, under the Patronage of his Royal Highness the Duke of Sussex.

IT is a fact too well known to require any elucidation, that the medical practice of our professional brethren on the opposite side of the English channel, is much more simple and mild than the mode of treatment usually adopted, under similar circumstances, by British practitioners. I will not stop to inquire into their relative superiority and success; nor whether the measures which are deemed expedient and salutary, in our insular situation, would be generally applicable to the natives of France. It is probable, however, that, in presuming to censure and ridicule what may appear to us very inert proceedings, we have taken too confined a view of the subject. For, in consequence of their more abstemious habits, added to their more temperate climate, the inhabitants are less plethoric, and less prone to diseases of high action, than we are; and, consequently, the same energetic measures, which are imperiously called for in the severe form of inflammatory ailments amongst us, would be ill adapted to the less sanguine and more relaxed constitution of our continental neighbours. I believe, too, it will scarcely be disputed, by those who are competent to form a correct judgment of their medical topography, that the Gallic nation is, upon the whole, a more healthy race than the Sons of Albion. One, among the various causes usually assigned in explanation of this greater freedom from disease, seems to me to have been too much overlooked; because, by resorting to the prophylactic means they adopt, I am inclined to believe that one grand source of morbid affections would at least be greatly lessened, if not altogether removed. I allude to the studious attention which is paid by the French of both sexes to the preservation of the alimentary canal in a regularly open state, by the constant and *almost daily* exhibition of domestic glysters. We have not now to learn, that many anomalous symptoms, and some very formidable maladies, may be fairly imputed to the bowels being suffered to remain habitually constipated. Nor is it necessary for me to enlarge upon the utility of purgatives in various diseases; a subject which has been so ably treated by Dr. Hamilton of Edinburgh; who has, by the most legitimate reasoning, founded upon incontrovertible facts, shown the importance in the prevention and cure of diseases which attaches to the removal of accumulated fæces, and the too long detention of indigestible colluvies, and vitiated intestinal secretions. So general has been the influence of his labours upon this interesting topic, that the free use of remedies of this description, *under actual disease*, constitutes a prominent feature of the present system of medical practice.

Such, however, is the almost unconquerable aversion which many individuals, (the inactive state of whose bowels requires some assistance,) entertain to the frequent swallowing of aperient medicines, that, rather than submit to the irksome and nauseating draught, they prefer subjecting themselves to all the inconvenience and dangers even of habitual costiveness. Independently, indeed, of this objection, the frequent introduction of cathartic ingredients, (which, by repetition, lose much of their efficacy,) into the stomach, that has often no share in the existing torpor, cannot be altogether free from injurious effects. This organ, being excited into unnatural movements, acquires, by degrees, a morbid irritability; and, becoming impatient of the stimulus of food, propels it forward into the duodenum, before it has undergone the changes which that important viscus is designed to elaborate upon the ingesta. In this manner it is probable that the stomach, by this repeated excitement, is made gradually to participate in that train of diseased action which primarily was confined exclusively to the lower portion of the alimentary tube. In the pregnant state, all kinds almost of opening medicines which are attempted to be given by the mouth are commonly productive of great distress, and are soon rejected by the stomach; the repetition of which tends still more to increase the irritability of that organ; whilst, on the contrary, an appropriate enema not only answers the purpose of preserving the bowels regular, but, by giving a peristaltic motion to their lower extremity, serves to counteract the disposition to the inverted action of the superior portion of the alimentary tube. Nor is this all; for, by removing accumulated and hardened fæces, which purgatives do not always accomplish, one grand cause of tenesmus, and pain in the abdomen, dysuria, hæmorrhoids, and even abortion, is obviated.

By the frequent employment of cathartics, either in a liquid or pilular form, the liver, the pancreas, and the glands of the mucous membrane of the intestines, are probably, directly or indirectly, through the intervention of their excretory ducts, kept in a constant state of fretfulness. To this may be added, that, in the majority of instances, abdominal accumulation takes place in the large, and but seldom in the small, intestines. On this account, it is clear that enemas, properly administered, by applying themselves immediately to the seat of the disease, afford the most likely means of relief, and without, too, the risk of entailing mischief upon the parts to which I have just adverted. These considerations, added to others of great weight which might be adduced, constitute a strong ground for preferring the French, before the prevailing English, mode of practice. In short, few other arguments need be advanced, I presume, to convince the public of the advantages that would result from the adoption of the continental usage, could a more convenient apparatus be procured for effecting the desired purpose. When once the comfort and benefit were fully experienced, every difficulty arising from the novelty of the measure would speedily vanish, and the practice would become as general as it would be found useful.

In the discharge of my professional duties, I have, in common with the rest of my brethren, had frequent cause to lament the inefficacy, and, in many instances, the impracticability of the means commonly resorted to for the exhibition of an enema, in cases where the relief, nay, the very existence of my patient was at stake. A proper pipe is not always at hand, and, even when it can be commanded, there is oftentimes as great an obstacle in procuring a bladder fit for the purpose: when even both are obtained and made ready for use, the latter not unfrequently becomes lacerated and unserviceable at a moment when the patient has, not without much persuasion, been prevailed upon to submit to the operation, from an assurance only of the great benefit which it would afford. Such an accident has repeatedly occurred to my observation; and, when it did happen, proved equally distressing to the patient, and mortifying and embarrassing to the surgeon; and, I may add, that the large pewter syringe, which has been made to supersede the use of the common fistula armata, or armed injection pipe, is by no means an easy, agreeable, or efficient, mode of administering domestic glysters.

In the hope of being able to obviate these difficulties, and to render a practice which challenges so many advantages more convenient and applicable at all times, and on every emergency, I thought I could not devote my time and study better than in contriving a manageable apparatus for this express purpose. After a great variety of experiments and alterations, I have at length the honor to submit to the approbation of my professional brethren an apparatus which, I

trust, will be found to embrace every requisite which can be desired in an instrument of this description. I have not only tried it myself with perfect satisfaction, but have the liberty also to refer for a confirmation of its utility, and for the ease and effect with which it can be used, to many of the most eminent physicians and surgeons in this metropolis, under whose direction it has been most successfully employed.

4, *Great Ryder-street, St. James's-square;*

*August*, 1818.

*Description, Delineation, and Method of using the Self-acting Patent Injecting or Glyster Apparatus.*

The capital A represents the metallic case, or receiver, made of a convenient shape, and of an ordinary size, capable of containing a pint of fluid. It has two perforations on the upper part; the one marked *a*, through which the prepared injection is to be introduced; the other aperture *b*, into which the air, or forcing syringe, is fitted; *c* denotes a stop-cock, which is connected on one side with the receiver A, and on the other with the ivory pipe *d*.

The following is the method of preparing the apparatus for use:—

First, take out the metallic screw made to close the aperture *a*, and introduce, through its circular opening, the requisite quantity of prepared injection, of a proper degree of heat. The stopper must be again replaced, and firmly screwed down, so as effectually to prevent the escape of air. Then, taking hold of the handle of the forcing syringe at *b*, the piston must be moved up and down, in pretty rapid succession, to its full extent, until a resistance is opposed to its descent; which will require about thirty strokes of the piston.

In this state, the apparatus being charged with the injection, and with condensed air, may be considered fit for use: previously, however, the handle of the forcing syringe may, if found inconvenient, be unscrewed and removed out of the way.

The ivory pipe is to be introduced in the usual manner; when, by gently turning more or less the stop-cock, the contents will, by the pressure of the atmospheric air accumulated on the surface of the liquid, be projected through the ivory conducting pipe, with as much force and as large a stream as the judgment of the operator, or the feelings of the patient, may suggest.

Substituting the flexible tube, B, in lieu of the ivory pipe *d*, the patient, by placing himself and the apparatus in a convenient situation, will be enabled, with the greatest facility and effect, to administer a glyster to himself, without the presence and aid of a second person.

## Figures and Tables

**Figure f1:**